# Human primary endothelial cells are impaired in nucleotide excision repair and sensitive to benzo[a]pyrene compared with smooth muscle cells and pericytes

**DOI:** 10.1038/s41598-019-49953-w

**Published:** 2019-09-24

**Authors:** Joana M. Kress, Lorella Di Dio, Larissa Heck, Alessandra Pulliero, Alberto Izzotti, Kathrin Laarmann, Gerhard Fritz, Bernd Kaina

**Affiliations:** 1 Institute of Toxicology, University Medical Center, Mainz, 55131 Germany; 2 Department of Health Sciences, University of Genoa, Genoa, 16132 Italy; 3 Hospital Policlinico San Martino, University of Genoa, Genoa, 16132 Italy; 4 Institute of Toxicology, Medical Faculty, Heinrich Heine University Düsseldorf, Düsseldorf, 40225 Germany

**Keywords:** Apoptosis, Cardiovascular genetics

## Abstract

The endothelium represents the inner cell layer of blood vessels and is supported by smooth muscle cells and pericytes, which form the vessel structure. The endothelium is involved in the pathogenesis of many diseases, including the development of atherosclerosis. Due to direct blood contact, the blood vessel endothelium is inevitably exposed to genotoxic substances that are systemically taken up by the body, including benzo[a]pyrene, which is a major genotoxic component in cigarette smoke and a common environmental mutagen and human carcinogen. Here, we evaluated the impact of benzo[a]pyrene diol epoxide (BPDE), which is the reactive metabolite of benzo[a]pyrene, on the three innermost vessel cell types. Primary human endothelial cells (HUVEC), primary human smooth muscle cells (HUASMC) and primary human pericytes (HPC) were treated with BPDE, and analyses of cytotoxicity, cellular senescence and genotoxic effects were then performed. The results showed that HUVEC were more sensitive to the cytotoxic activity of BPDE than HUASMC and HPC. We further show that HUVEC display a detraction in the repair of BPDE-induced adducts, as determined through the comet assay and the quantification of BPDE adducts in post-labelling experiments. A screening for DNA repair factors revealed that the nucleotide excision repair (NER) proteins ERCC1, XPF and ligase I were expressed at lower levels in HUVEC compared with HUASMC and HPC, which corresponds with the impaired NER-mediated removal of BPDE adducts from DNA. Taken together, the data revealed that HUVEC exhibit an unexpected DNA repair-impaired phenotype, which has implications on the response of the endothelium to genotoxicants that induce bulky DNA lesions, including the development of vascular diseases resulting from smoking and environmental pollution.

## Introduction

The inner layer of blood vessels is lined with more than 10 billion endothelial cells, which regulate, among other features, coagulation and blood pressure and play an important role in wound healing^1^. Smooth muscle cells, which surround the larger vessel structures, directly interact with the endothelium and are connected with it through a complex signalling system. Together with pericytes, endothelial cells and smooth muscle cells are essential for angiogenesis^2–4^. Pericytes are mainly found on capillaries and post-capillary venules, where they are in direct cell-cell contact with the endothelial cell layer^5,6^. Based on the structure of blood vessels, it is reasonable to hypothesize that endothelial cells are primarily exposed to genotoxins that enter the blood stream and could thus be considered the first target of endogenous and exogenous systemically available DNA-damaging agents. Indeed, genotoxic agents, regardless of whether they are of endogenous or exogenous origin, target the endothelium, as demonstrated by the finding of DNA adducts in atherosclerotic lesions^7^. Furthermore, the level of genotoxic damage has been demonstrated to be predictive for patient’s survival^8^.

The genotoxic metabolite benzo[a]pyrene-7*R*,8*S*-dihydrodiol-9*S*,10*R*-epoxide (BPDE) is the active form of the widely distributed polycyclic aromatic hydrocarbon benzo[a]pyrene (B[a]P), which is found in tobacco smoke, roasted meat, environmental pollutants and many products obtained from incomplete combustion^9^. It has been classified by the IARC as a group 1 human carcinogen. B[a]P forms BPDE through a three-step activation process catalysed by human cytochrome P450 enzymes, notably CYP1A1 and CYP1B1, and by epoxide hydrolase. Approximately 10% of B[a]P is converted to BPDE^10^. The U.S. population has a daily intake of approximately 2.2 µg of B[a]P^9^, which corresponds to about 220 ng BPDE. How much of this is systemically available and reaches the target organ is an open question. BPDE is highly carcinogenic and can bind to the exocyclic *N*2 position of guanine to form bulky adducts in a dose-dependent manner^11^. These bulky adducts are substrates for nucleotide excision repair (NER), which protects against the toxic, mutagenic and carcinogenic effects of B[a]P^12–14^. Data on the repair of BPDE adducts and their biological consequences in human endothelial cells are not available.

The wide environmental distribution of B[a]P and the existence of defence systems, including DNA repair, encouraged us to question the extent to which the various blood vessel cell types are protected against BPDE and the differences in their sensitivity to DNA insults resulting from BPDE exposure. To address these questions, we compared the responses of human primary endothelial cells (HUVEC), human primary smooth muscle cells (HUASMC) and human primary pericytes (HPC) to BPDE. Furthermore, guided by our previous study, which demonstrated that some DNA repair genes show an adaptive upregulation in response to BPDE treatment^15^, we studied the basal expression level of NER proteins/enzymes and the expression of DDB2 and XPC following BPDE exposure. Our findings revealed that HUVEC are more sensitive to BPDE than HPC and HUASMC and display an unexpected DNA repair-impaired phenotype.

## Results

### HUVEC are more sensitive to BPDE than HUASMC and HPC

**Figure 1:**
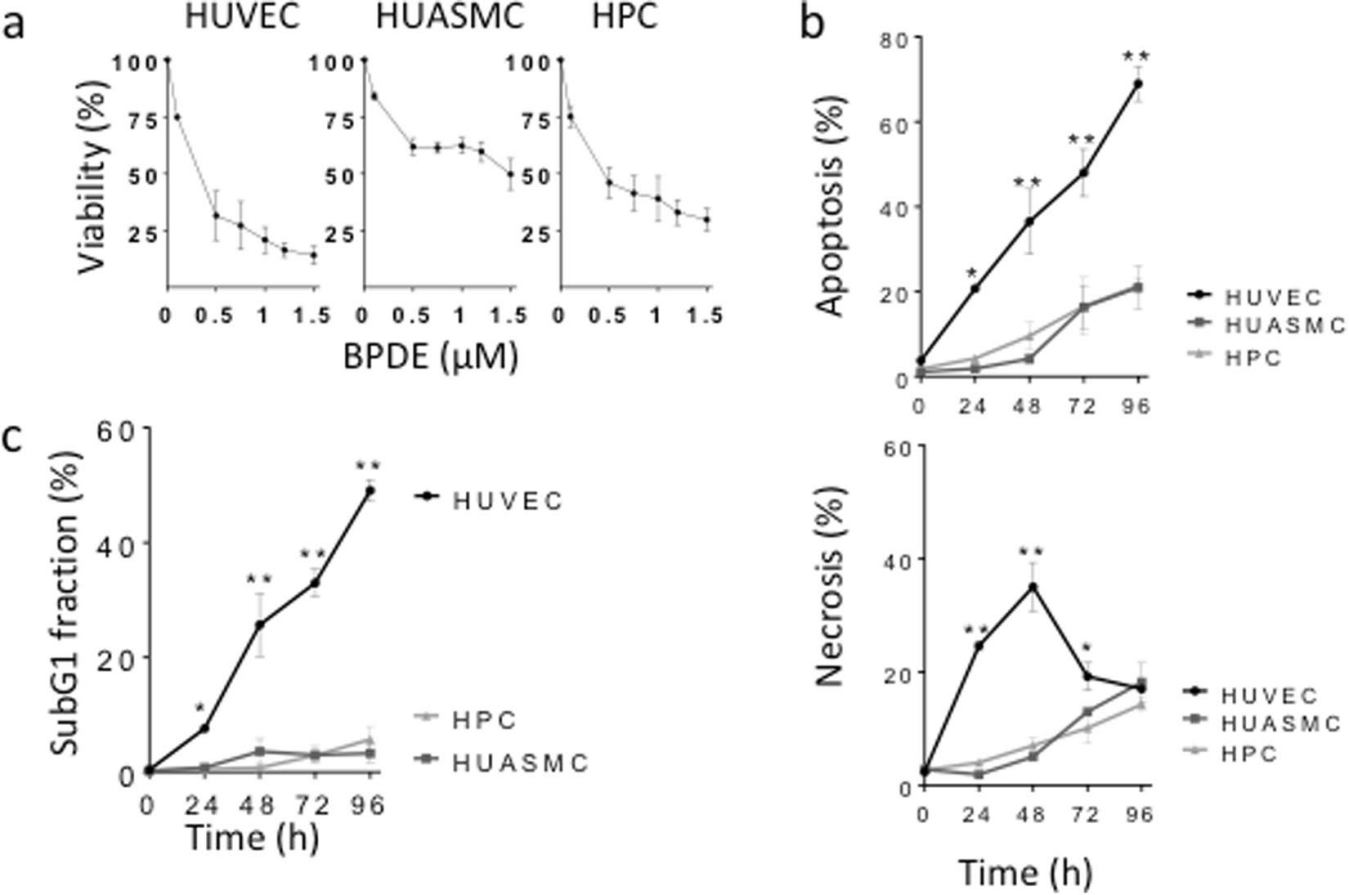
Cytotoxicity induced by BPDE in human vascular cells: HUVEC, HUASMC and HPC. (**a**) Dose-dependent effect of BPDE on viability, as measured in the MTT assay 72 h after treatment with BPDE. Viability is expressed as percentage relative to the non-treated control. (**b**) Induction of apoptosis and necrosis following 1.5 µM BPDE, measured at different times after treatment and determined by annexin V/PI flow cytometry. (**c**) Apoptosis measured by subG1 quantification at different times after treatment with 1.5 µM BPDE. All data are the mean ± SEM of at least three independent experiments.

To compare the effect of BPDE on vascular cells, we first determined the cytotoxicity of BPDE in primary HUVEC, HUASMC and HPC through the MTT viability assay and apoptosis and necrosis measurement through annexin V/PI flow cytometry. The viability assay revealed that HUVEC are more sensitive to treatment with BPDE than HUASMC and HPC (Fig. 1a). Specifically, a dose of 0.5 µM BPDE reduced the viabilities of HUVEC, HUASMC and HPC to 31, 61 and 46%, respectively. The apoptosis and necrosis levels after treatment with 1.5 µM (Fig. 1b) and 0.75 µM BPDE (Supplementary Material, Fig. S1A) were determined, and the results showed that HUVEC exhibited the highest levels of apoptosis and necrosis among the three tested cell types, which was observed at all tested post-exposure times. The high sensitivity of HUVEC to BPDE was confirmed by measuring the rate of apoptosis through subG1 flow cytometry, demonstrating a dramatic difference in apoptosis rate between HUVEC on the one hand and HUASMC and HPC on the other (Fig. 1c).

**Figure 2:**
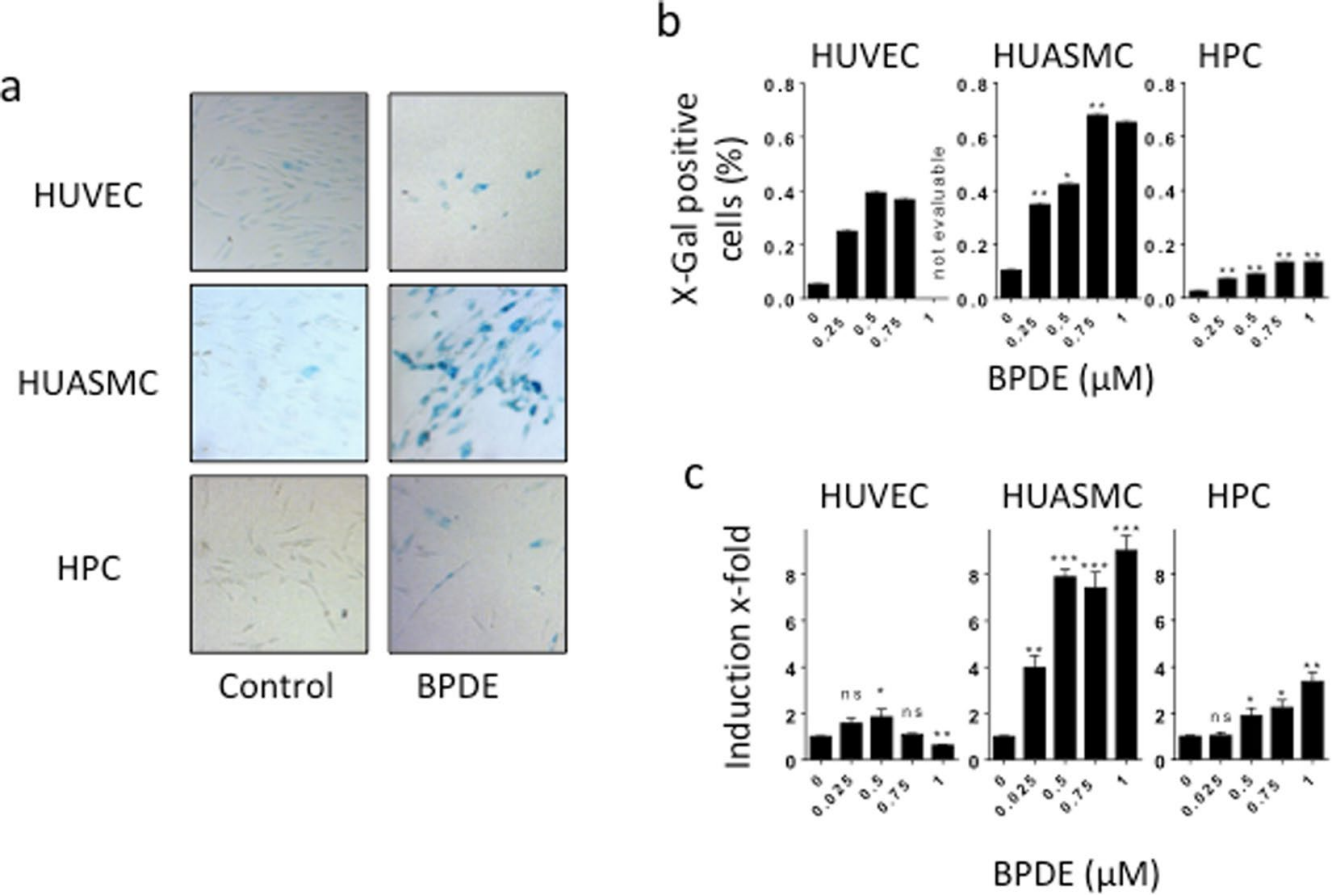
Induction of senescence after BPDE treatment in HUVEC, HUASMC and HPC. (**a**) Cellular senescence 96 h after treatment with BPDE visualised by β-galactosidase staining. Representative images of β-galactosidase activity (blue stained cells) in non-treated controls and after exposure to 0.75 µM BPDE. (**b**) Amount of senescent cells upon treatment with 0.25, 0.5, 0.75 and 1 µM BPDE. The highest dose of 1 µM BPDE could not be evaluated in HUVEC because of strong cytotoxicity induced in these cells. Data shown are the mean ± SEM of three independent experiments; a minimum of 1000 cells were counted per experiment. Significance was calculated compared to HUVEC. (**c**) Level of p21 mRNA 24 h after exposure of the indicated doses of BPDE. Data are the mean ± SD of three independent experiments, each performed in duplicate.

To ascertain whether the cells undergo premature senescence following BPDE treatment, we measured the activity of senescence-associated β-galactosidase. Representative ß-gal stainings are shown in Fig. 2a. The quantification of the results (Fig. 2b) revealed that HUVEC and HUASMC displayed cellular senescence induction already after exposure to a low BPDE dose (0.25 µM). The maximum percentages of β-galactosidase-positive HUASMC and HUVEC were 67% and 40%, respectively. In contrast, HPC showed only a weak induction of premature senescence, as demonstrated by the finding that less than 14% displayed β-galactosidase-positive staining. We also assessed cell cycle progression and the induction of p21, which is involved in the regulation of senescence, in HUVEC, HUASMC and HPC following BPDE treatment. In all cell types, the G1 fraction declined and S and G2/M increased following treatment, indicating S blockage and G2 accumulation (Supplementary Material, Fig. S1B). Regarding p21, the highest level was observed in HUASMC, corresponding to the high senescence level. It was less induced in HUVEC and marginally only at the highest dose in HPC (Fig. 2c).

### Single-strand breaks (SSBs) accumulate in HUVEC after exposure to BPDE

**Figure 3:**
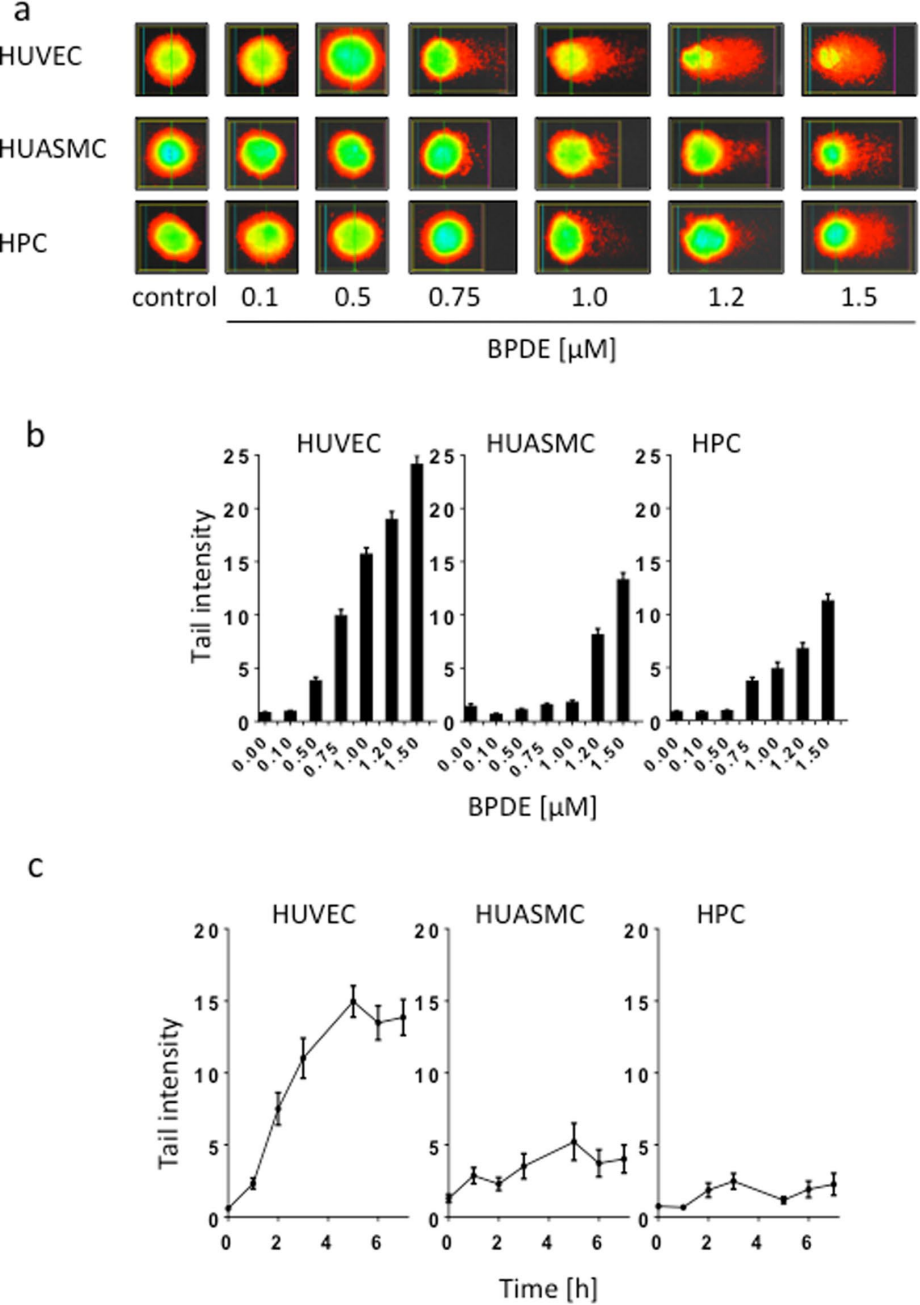
Genotoxic effects of BPDE in HUVEC, HUASMC and HPC. (**a**) Representative images of comets in HUVEC, HUASMC and HPC treated with the indicated doses of BPDE and measured 2.5 h after addition of BPDE to the medium. (**b**) Tail intensity determined by the alkaline comet assay as a function of dose of BPDE. Cells were fixed and stained 2.5 h after treatment. Each measure point represents the mean of three independent experiments ± SEM; a minimum of 50 cells were measured per experiment. (**c**) Tail intensity in the alkaline comet assay after treatment with 0.5 µM BPDE as a function of time. Each measure point represents the mean of three independent experiments ± SEM. Cells were embedded in agarose, lysed and subjected to electrophoresis as described in Material and Methods. Image analysis and calculation of tail intensities were performed with Comet IV software.

To determine the genotoxic effects induced by BPDE in the investigated cell types and the repair of BPDE adducts, we used the alkaline comet assay to visualize the amount of SSBs in the DNA. It is generally accepted that SSBs, which are observed after treatment with genotoxins that induce bulky adducts, represent intermediates of the NER process. Thus, during NER, incisions are made 5′ and 3′ from the lesion by the NER endonucleases ERCC1/XPF and XPG, respectively. The nucleotide fragment containing the damage is removed, and the resulting gap is sealed by repair synthesis and completed by ligation through ligase I and possibly also ligase III. If not immediately sealed, these repair intermediates give rise to a reduction in the molecular weight of DNA that can be detected through the comet assay. Representative examples of single cells analysed in the alkaline comet assay from the untreated control populations and the populations after BPDE treatment are shown in Fig. 3a. The quantification of the migrated DNA revealed that HUVEC displayed the highest level of SSBs, and this finding was obtained with all doses of BPDE. Compared with primary HUVEC, the other cell populations HUASMC and HPC revealed much lower levels of SSBs, particularly after exposure to the lower BPDE doses. Thus, with a dose of 0.5 µM BPDE, HUASMC and HPC did not display a significant increase above the control, while in HUVEC the level was significantly enhanced above the non-treatment control level (p < 0.01) (Fig. 3b).

To assess whether the induction of SSBs and the start of the repair process in HUVEC show differences compared with the other cell types, we performed a time course experiment. During the first 7 h of treatment with 0.5 µM BPDE, SSB induction and accumulation were clearly observed in HUVEC, whereas the responses observed in HUASMC and HPC did not reach the same levels (Fig. 3c). Based on this data, we conclude that in HUVEC, BPDE adducts are recognized and incision occurs. Although this happens, adducts are only incompletely repaired and repair patches are not sealed. Therefore, gaps are left in the DNA resulting in persisting SSBs that were detected in the comet assay. In contrast, in HUASMC and HPC, NER occurs nearly to completion, and thus, the NER intermediates do not give rise to a substantial accumulation of SSBs.

### BPDE-DNA adducts are not completely removed from DNA in HUVEC

**Figure 4:**
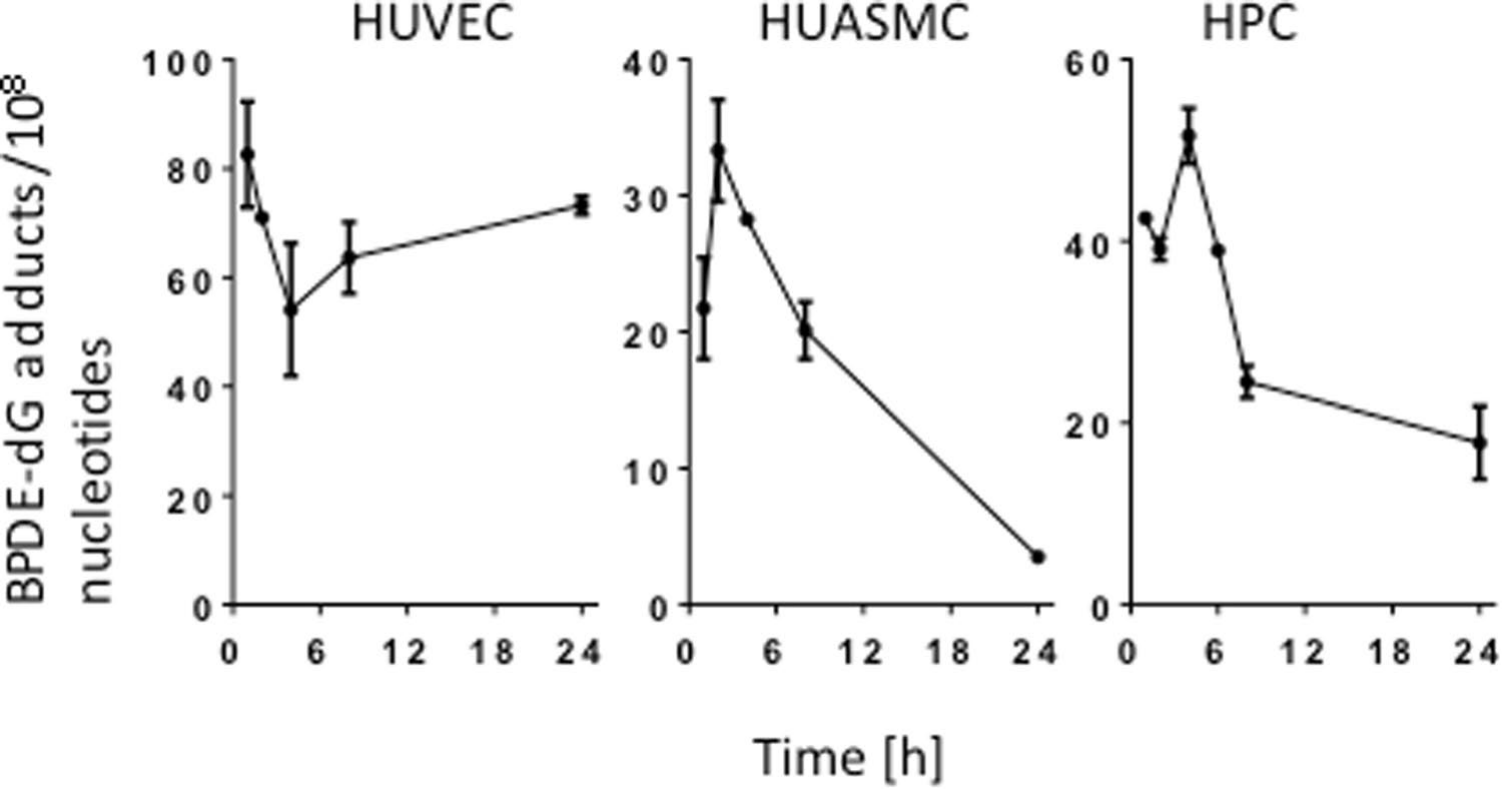
BPDE-adduct levels in HUVEC, HUASMC and HPC. BPDE adducts (per 10^8^ nucleotides) in HUVEC, HUASMC and HPC cells following exposure to 0.5 µM BPDE at different times after treatment. Adducts were measured by the ^32^P-postlabeling method as described in Material and Methods. Data shown are the mean of the results obtained in two independent experiments.

To substantiate the conclusions drawn from the comet assay, we set out to directly determine the removal of BPDE adducts from DNA. Using the post-labelling method^16^, we measured the amount of BPDE adducts per 10^8^ nucleotides over time. The results showed that the adduct level remains unchanged in HUVEC, whereas in HUASMC and HPC a decrease in the adduct level was observed during a 24 h post-exposure period (Fig. 4; for representative post-labelling images, see Supplementary Fig. S2). The data indicate that BPDE adducts are not removed from DNA in HUVEC. We should note that the amount of adducts was generally lower in HUASMC and HPC than in HUVEC, which can be explained by the higher repair capacity of these cell types, resulting in removal of adducts within the treatment and 1 h post-exposure period. Of note, BPDE has a short half-life in medium and thus causes adduct formation within minutes after the initiation of treatment. The high adduct levels observed in HUVEC up to 24 h after pulse treatment supports the conclusion that HUVEC are defective or, at least, impaired in NER.

### Expression of NER proteins

**Figure 5:**
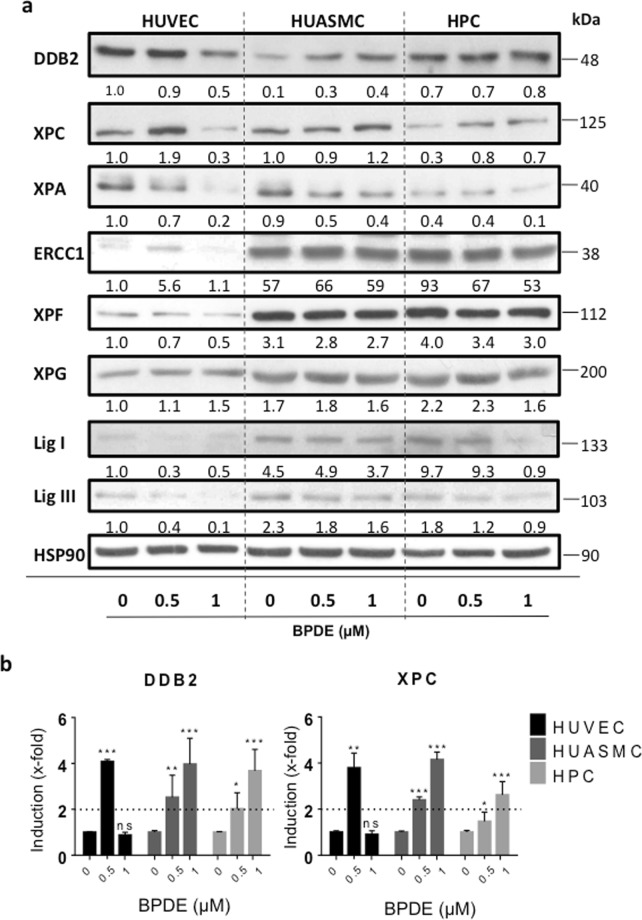
NER protein expression in HUVEC, HUASMC and HPC not treated and treated with BPDE. (**a**) Dose-dependent expression of NER core proteins was analyzed by immuno-detection in total cell extracts 24 h after exposure to 0, 0.5 and 1 µM BPDE. HSP90 was used as loading control. HUVEC, HUASMC and HPC extracts were loaded onto the same blot in each experiment in order to have a direct comparison. The same extracts were used in different immunoblots for the indicated proteins. The loading control (HSP90) shown in the figure is a representative example. The numbers below each blot for the corresponding repair protein indicate the relative expression level, in which HUVEC control was set to 1. Quantitative analysis of blot intensity was done with ImageJ and related to the HSP90 control in the same blot. Expression data of a representative experiment are shown. (**b**) mRNA expression of DDB2 and XPC in HUVEC, HUASMC and HPC following treatment with 0, 0.5 and 1 µM BPDE. Cells were harvested 24 h after treatment and the expression was determined by RT-PCR. Data show the mean ± SD of three independent experiments. The relative mRNA expression level of the non-treated control was set to 1.0. The difference to the control was not significant (ns) or significant (p = 0.05 *0.01**0.001***).

To further substantiate the conclusion that the high sensitivity of HUVEC to BPDE is due to impaired NER, we compared the expression levels of core NER proteins among HUVEC, HUASMC and HPC. A Western blot analysis revealed that the cell types display significant differences in the basal NER protein expression (Fig. 5). Thus, the expression of the NER recognition proteins DDB2, XPC and XPA was not reduced in HUVEC compared with HUASMC and HPC. However, HUVEC expressed a clearly lower level (both basal and after BPDE treatment) of the NER proteins ERCC1, XPF and ligase I, which play key roles in the NER pathway, catalysing the incision 5′ to the adduct (the complex ERCC1/XPF acting as NER endonuclease) and the ligation of newly synthesized fragments, respectively. Ligase III was reported to be able to replace ligase I and supporting NER^17^, and thus it is interesting to see that ligase III is also expressed at lower level in HUVEC than in HUASMC and HPC. The basal expression of the NER polymerase Pol ε was also slightly reduced. This was not the case for Pol δ (Supplement Fig. S3). Interestingly, 24 h after treatment with 1 µM BPDE, DDB2 and XPC were upregulated at protein level in HUASMC and HPC, respectively, but not in HUVEC. In contrast to this, the response of HUVECs to BPDE was characterized by a substantial decline in the DDB2 and XPA levels (Fig. 5a). These data might be explained by the high adduct levels observed in HUVEC, which may inhibit transcription and thus attenuate the induced response, which is a specific attribute following BPDE treatment as shown previously with human fibroblasts treated with BPDE doses above 0.75 µM^15^. This supposition gains support from expression studies on mRNA level, where we observed after treatment with 1 µM BPDE lack of induction of DDB2 and XPC in HUVEC, while in HUASMC and HPC the level was significantly enhanced (2 up to 4-fold). However, with a low dose of 0.5 µM we observed DDB2 and XPC mRNA induction also in HUVEC (Fig. 5b). This is compatible with the notion that non-repaired adducts block the BPDE-induced DDB2/XPC response in HUVEC, and thus further attenuate the repair of adducts.

### Increased DNA damage response (DDR) in HUVEC after exposure to BPDE

**Figure 6:**
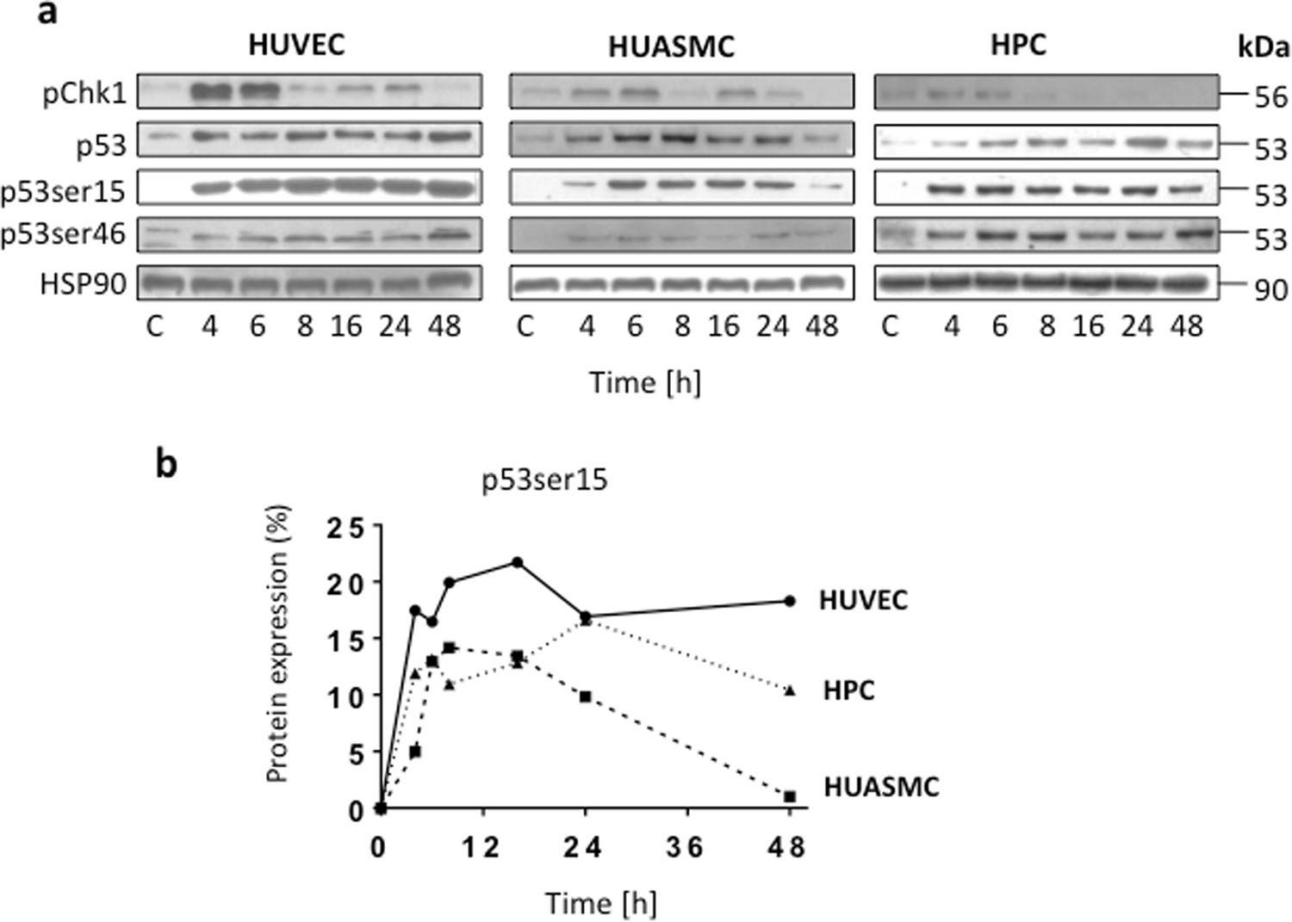
Level of non-phosphorylated and phosphoryated p53 and pChk1 in HUVEC, HUASMC and HPC. (**a**) The levels of pChk1, p53 and p53ser15/46 were analyzed in Western blots in total cell extracts at various time points after treatment with 1 µM BPDE. HSP90 was used as loading control. Immunostainings were performed with the same cell extracts that were used for separate blots. Representative blots are shown. (**b**) Quantification of p53ser15 in HUVEC, HUASMC and HPC. Data are related to the level in HUASMC measured 48 h after treatment, which was set to 1.

To analyse whether the DDR is affected in HUVEC, we determined the protein levels of pCHK1, p53, p53^ser15^ and p53^ser46^ in the three cell types at several times after BPDE treatment. CHK1 activation was detected 4 and 6 h after exposure, and the strongest activation level was observed in HUVEC (Fig. 6a), which agrees with the high BPDE adduct level found in these cells. The activation of p53 (as determined by its stabilization) was clearly detected in all three cell types, and we also observed p53 phosphorylation at serine 15 and serine 46. The highest level of activated p53 (p53^ser15^) was consistently observed in HUVEC (the quantified results are shown in Fig. 6b), which supported the notion that unrepaired DNA adducts in HUVEC provide a strong and sustained signal for p53^ser15^ activation.

## Discussion

The endothelium, which lines all blood vessels in the body and is supported by the underlying smooth muscle cells and pericytes that form the vessel structure, can be considered an organ^18^. Various studies have attempted to define the endothelium as a barrier that protects solid tissues against harmful substances present in blood^19^. A similar well-accepted phenomenon has been ascribed to the brain, where the blood-brain barrier fulfils a protective function^20^. Further studies have investigated the protection induced by the endothelium and the barrier function of this organ in the cornea^21^ and the thymus^22^. A healthy endothelium maintains important cardiovascular functions, e.g., the regulation of blood pressure, coagulation and angiogenesis, and endothelial impairment or dysfunction has been shown to be associated with several diseases, e.g., atherosclerosis, diabetes and hypertension^1,18^. Therefore, the protection of the endothelium, which constitutes a single-cell layer inside the blood vessels, appears to be of utmost importance.

Genotoxic agents, regardless of whether they are endogenously formed or taken up orally or via other routes, become systemically available through their presence in blood, and thus, their first contact occurs with the endothelium. Because the endothelium is the primary target of systemically available genotoxins, we initially hypothesized that endothelial cells are particularly well equipped with DNA repair functions that protect them against genotoxic substances present in blood. To prove this working hypothesis, we studied the genotoxic effect of BPDE, which is the activated form of benzo[a]pyrene - a widely distributed environmental and tobacco smoke genotoxin^23^ and a well-known carcinogen^24^. It has previously been shown that benzo[a]pyrene induces DNA damage in HUVEC^25^ and that vascular cells have the capacity to metabolize benzo[a]pyrene via cytochrome P4501A1, which is upregulated *in vitro* in HUVEC^25^ and SMC^26^ and *in vivo* in the rat aortic endothelium^27^, or *via* cytochrome P4501B1, which was found in mice pericytes^28^. However, there are no data available regarding the cytotoxic response and the repair of BPDE-induced DNA adducts in endothelial cells, particularly in comparison with other vessel cell types. Throughout this study, we used well-defined primary cell pools, measured the response of HUVEC to BPDE in comparison with primary smooth muscle cells and primary pericytes. Contrary to our expectation, the results showed that HUVEC are more sensitive than HUASMC and HPC to the cytotoxic, apoptotic and necrosis-inducing effects of BPDE. However, HUVEC were not more sensitive than HUASMC and HPC regarding the end-point cellular senescence, as measured by SA-ß-gal, which was clearly induced in all cell types by BPDE.

BPDE forms bulky lesions in DNA by binding to the exocyclic *N*2-position of guanine, and this adduct is subjected to repair through NER^29^. Our data revealed that the high sensitivity of HUVEC to the cytotoxicity of BPDE is due to high and persistent BPDE adduct levels and high amounts of repair intermediates (notably SSBs), as demonstrated through the comet assay. The post-labelling experiments confirmed this data and showed significantly less repair of BPDE adducts over time. Thus, the adduct level in HUVEC remained constant over a 24 h post-exposure period, while in HUASMC and HPC there was a decline with about 85 and 60% removal of induced adducts, respectively. These findings prompted us to measure the expression levels of key repair proteins. An expression-based screening of NER proteins revealed that the levels of ERCC1, XPF and ligase I were clearly lower in HUVEC than in HUASMC and HPC. The ERCC1/XPF complex exerts endonuclease activity by cleaving the DNA 5′ from the lesion, while cleavage 3′ from the lesion is catalysed by the endonuclease XPG^12^. Since this protein is expressed in HUVEC, we infer that incisions through XPG surrounding the lesion are made, but the repair process is not complete as ERCC1/XPF incisions are not made, resulting in the accumulation of repair intermediates (SSBs) in the DNA. The very low level of ligase I further supports the accumulation of repair intermediates. In summary, the low ERCC1/XPF and ligase I levels and incomplete NER observed in HUVEC explains the higher level of SSBs detected in HUVEC compared with HUASMC and HPC through the alkaline comet assay. This hypothesis also explains the finding that adducts are not removed from DNA. Thus, incisions are made by XPG, but the fragment containing the adduct cannot be removed because of lack of ERCC1/XPF mediated 5′ incision. In summary, all the observations reported in this manuscript lead to the conclusion that ERCC1/XPF is the rate-limiting NER step in HUVEC, preventing the efficient repair of BPDE adducts.

It is very likely that this conclusion also pertains to other bulky adducts induced by a plethora of genotoxins the endothelium is exposed to, but this hypothesis has to be confirmed in future studies. Whether impaired NER is a specific property of HUVEC or a phenotype that also pertains to other vascular endothelial cells also needs to be elucidated. We should note that the established endothelial cell line EA.hy926, which is often used for studies on endothelial function, represents only a surrogate cell system because it is a hybrid cell type in which the repair functions that are significantly reduced in HUVEC are likely complemented by the cell type used for hybridoma formation. Therefore, we used primary HUVEC throughout the study, and we also used pooled primary stocks to minimize donor variations.

Tobacco smoke containing benzo[a]pyrene is not only responsible for one third of all cancer cases, but also associated with a significant increase in cardiovascular diseases. Thus, it has been estimated that an individual who consumes less than five cigarettes per day^30^, which corresponds to an uptake of 29–190 ng of benzo[a]pyrene per day, has 1.5- and 2-fold higher risks for stroke and heart infarct, respectively^31^, and even living with a smoker significantly increases the risks of the non-smoking partner^32^. Although the causal link between smoking and cardiovascular diseases is well accepted, little is known regarding the factors leading to cardiovascular failure. Thus, ROS, chemical carcinogens, tumour promoters, particles and nicotine in tobacco smoke have been considered aetiological factors. A well-established theory claims that the primary reason for cardiovascular diseases lies in damage of the endothelium, which causes endothelial cell death followed by a local inflammatory response characterized by immune cell recruitment and activation and, in the late stage, plaque formation and rupture^33^. Tobacco smoke contains more than 60 well-defined carcinogens, some of which are highly genotoxic and carcinogenic^34^. Despite this knowledge, the most critical factor(s) in tobacco smoke and in the environment responsible for endothelial damage following uptake have not been identified. Because our study showed that endothelial cells have a decreased ability to repair BPDE-induced DNA adducts and display hypersensitivity to BPDE, it is conceivable that BPDE produced from benzo[a]pyrene metabolism in the endothelium causes significant DNA damage in this target organ, resulting in cell death and local vascular damage. Because smooth muscle cells and pericytes appear to be protected, the cell death limited to the endothelium will not necessarily result in cardiovascular failure, but if the regenerative process fails, it could result in a local lesion that promotes plaque formation.

Endothelial cells are exposed to many chemical stressors and it is important to understand their sensitivities and responses to these stress factors. Thus, further studies are warranted to assess the sensitivity and repair capacity of HUVEC compared to other cell types and even cancer cells, since systemically applied genotoxic drugs are often used in cancer chemotherapy. The studies might also be extended to radiation in order to better understand the radiation response of HUVEC^35^ and other vascular cell types.

The high cytotoxic response of HUVEC may be considered as a counteractive mechanism to tumor formation. Interestingly, hemangiosarcoma resulting from transformed endothelial cells is a highly aggressive, the blood vessel invading and rare malignancy in humans^36^, although it is frequently observed in dogs^37^. Whether hemangiosarcoma are more frequent in smokers than in the general population is, to our very best knowledge, not known. Nevertheless, it is conceivable that the impaired NER pathway in HUVEC represents a mechanism of cancer defense as damaged endothelial cells harbouring carcinogenic adducts are eliminated through apoptosis. Nevertheless, the failure of HUVEC to efficiently repair bulky DNA adducts and the related cytotoxic response might be a driving force in the genesis of vascular damage and cardiovascular diseases, including blood pressure deregulation and atherosclerosis.

## Methods

### Cell culture and chemicals

Studies were performed using pooled primary human umbilical vein endothelial cells (HUVEC, C-12203), human umbilical artery smooth muscle cells (HUASMC, C-12500) and human pericytes from placenta (HPC, hPC-PL, C-12980) purchased from PromoCell (Heidelberg, Germany). Cells were grown in the corresponding media from PromoCell (Heidelberg, Germany): Endothelial Cell Growth Medium 2 (Ready-to-use, C-22011), Smooth Muscle Cell Growth Medium 2 (Ready-to-use, C-22062) and Pericyte Growth Medium (Ready-to-use, C-28040) with addition of 1% penicillin/streptomycin (Gibco). Cells were routinely cultured under nitrogen atmosphere (7% O_2_, 5% CO_2_) and passaged twice a week for a maximum of 15 passages using trypsin (Gibco). BPDE was purchased from BIU Biochemical Institute for Environmental Carcinogens, Prof. Dr. G. Grimmer-Stiftung (Grosshansdorf, Germany). BPDE was dissolved in water-free THF/5% trimethylamine (1 mM) and stored at −80 °C. To avoid hydrolysis of the epoxides, dilutions from the stock solution were always prepared fresh immediately before incubation.

### Determination of apoptosis/necrosis and metabolic competence

Apoptosis and necrosis induction was determined by harvesting the cells at different time points after BPDE exposure and performing annexin V-FITC/PI double staining combined with flow cytometry. The protocol was essentially as described^38^. The metabolic competence of BPDE-exposed cells was determined by 3-(4,5-dimethyldiazol-2-yl)-2,5-diphenyltetrazolium bromide (MTT) assay as already described^39^. In all cases, experiments were repeated at least three times, mean values ± standard error of the mean (SEM) are shown.

### Determination of senescence

96 h after exposure to BPDE, cytochemical senescence detection was performed with the senescence β-Galactosidase Staining Kit (Cell Signaling) according to the manufacturer’s instruction. Quantification occurred as described^40^.

### Comet assay

After different time points of exposure to BPDE the alkaline comet assay was performed as previously described^41^.

### Preparation of protein extracts and western blot analysis

Whole-cell extracts were prepared by lysing the cells directly in 1 × sodium dodecyl sulphate-polyacrylamide gel electrophoresis sample buffer and subsequent sonification. Antibodies were diluted 1:200–1:1000 in 5% bovine serum albumin (BSA) or 5% milk, 0.1% Tween-TBS and incubated overnight at 4 °C. Anti-p53, anti-HSP90, anti-DDB2, anti-XPC, anti-XPA, anti-ERCC1, anti-XPF, anti-XPG, anti-Pol ε, anti-Pol δ, anti-Lig I and anti-Lig III antibodies were purchased from Santa Cruz Biotechnology (Dallas, TX, USA). Anti-pChk1, anti p53ser15 and anti-p53ser46 antibodies were obtained from Cell Signaling Technology (Danvers, MA, USA). The protein–antibody complexes were visualized by Pierce ECL Western Blotting Substrate (32106, Thermo Fisher).

### DNA isolation and ^32^P-postlabeling of BPDE-adducts

Genomic DNA was isolated by phenol-chloroform extraction as previously described^42^ and quantity and quality was evaluated by nano-spectrophotometry (Nanodrop, Thermo Fisgher Sci., Waltham, MA, USA). ^32^P-postlabeling was performed according to previously published procedure^16^. Briefly, DNA (10 µg) was depolymerized by microccocal nuclease and spleen phosphodiesterase (Sigma) and lipophilic adducts extracted by water-saturated butanol. Butanol was evaporated by vacuum centrifugation and adducts labelled by T4 polynucleotide kinase (AB Analitica, Padua, Italy) using AT-gamma-^32^P (>6,000 Ci/mmol) (Perkin Elmer, Boston, MA, USA) as ^32^P donor. ^32^P-labelled adducts were resolved by multi-directional thin layer chromatography on polyethylenimine sheets (Macherey Nagel, Duren, Germany) performing washings in sodium phosphate buffers (1 and 1.7 M) and resolution runs in urea buffers (8.5 M, pH 8.5 and 3.5). Chromatographic sheets were dried under warm air and analyzed for emitted radiation by phosphorimager (^32^P imager, Packard, Meriden, CT. USA). Radioactive spots were quantified for emitted radiation, relative adduct labelling index calculated referring to normal nucleotides and results expressed as adducts per 10^8^ normal nucleotides. DNA free sample was used as sham control; a standard of authentic BPDE-*N*2-dG adducts (NCI repository, USA) was used as positive reference standard.

### RNA isolation, cDNA synthesis and real-time PCR

RNA isolation was performed with the RNA extraction kit peq gold RNA kit (peqlab, Erlangen, Germany). RNA concentrations were determined by measuring the absorbance at 260 and 280 nm. 1 µg of RNA was used for cDNA synthesis with the Verso cDNA Synthesis Kit (Thermo Fisher Scientific, Darmstadt, Germany). qPCR was performed by CFX96 Real-Time PCR Detection System (Bio-Rad Laboratories GmbH, Hercules, CA, USA). Gapdh and Actb served as internal housekeeping genes. Primer sequences are shown in Supplementary Table 1.

### Statistics and ethic statement

Data were statistically analyzed using Student’s unpaired two-sample *t*-test on the basis of a difference between sample means. Calculations were performed using the GraphPad Prism software (*P < 0.05, **P < 0.005, ***P < 0.0005). All methods were carried out in accordance with relevant guidelines and regulations. Primary cell stocks obtained from PromoCell (Heidelberg, Germany) were approved and generated according to the “German Federal Data Protection Act”.

## Supplementary information

Dataset 1
