# Supplementary material for: Human primary endothelial cells are impaired in nucleotide excision repair and sensitive to benzo[a]pyrene compared with smooth muscle cells and pericytes
Source: Sci Rep. 2019 Sep 24;9:13800. doi: 10.1038/s41598-019-49953-w (PMC6760230; doi:10.1038/s41598-019-49953-w)
Supplement: Supplementary file 1 — Dataset 1 [file 41598_2019_49953_MOESM1_ESM.pdf]

Supplementary material

Human primary endothelial cells are impaired in nucleotide excision repair and sensitive to benzo[a]pyrene compared with pericytes and smooth muscle cells

Joana M. Kress[1], Lorella Di Dio[1], Larissa Heck[1], Alberto Izzotti[2], Alessandra Pulliero[2], Kathrin Laarmann[3], Gerhard Fritz[3] and Bernd Kaina[1]

A

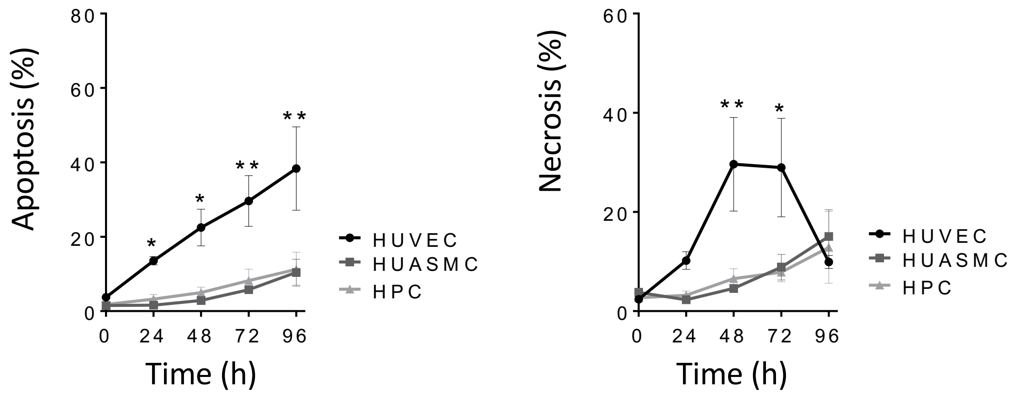

B

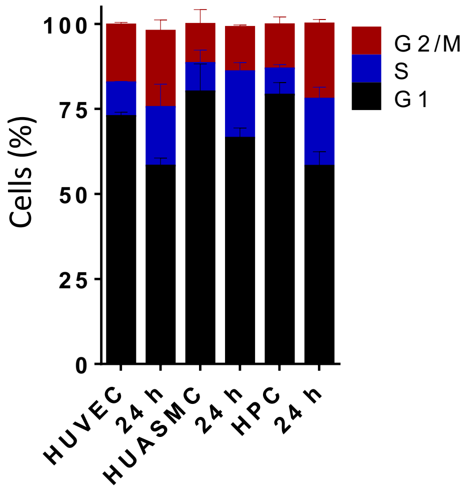

Figure S1: Apoptosis and necrosis and cell cycle distribution upon exposure to BPDE in human vascular cells (HUVEC, HUASMC and HPC).

A, Time-dependend apoptosis and necrosis following 0.75 µM BPDE treatment was determined by annexin V/PI double staining and flow cytometry.

B, Cell cycle distribution of non-treated cells and treated cells (0.75 µM BPDE for 24 h) was measured by propidium iodide staining and flow cytometry. Data shown are the mean ± SEM from three independent experiments.

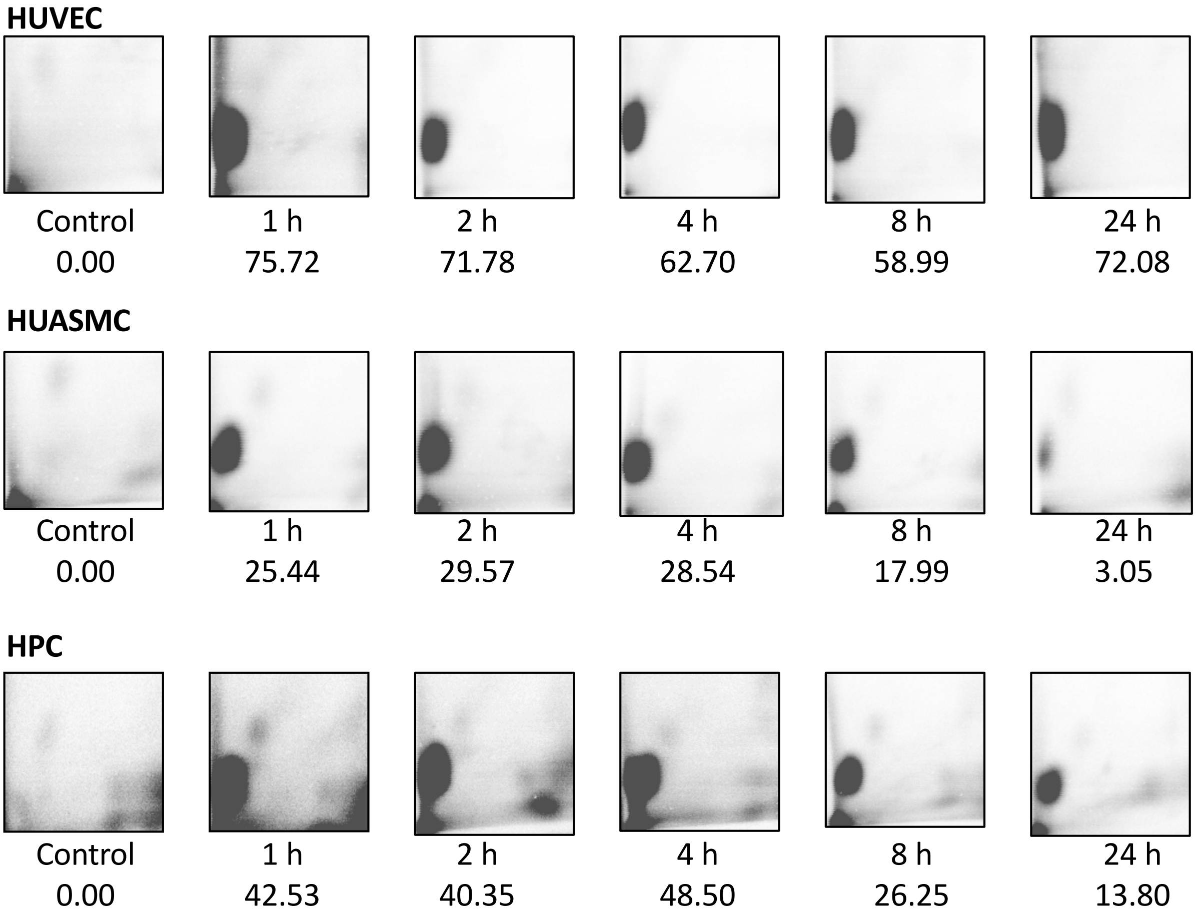

Figure S2: Time-dependend BPDE-adduct levels. BPDE-adducts per 10^8 nucleotides were determined 1, 2, 4, 8 and 24 h after exposure to 0.5 µM BPDE. Representative plots are shown.

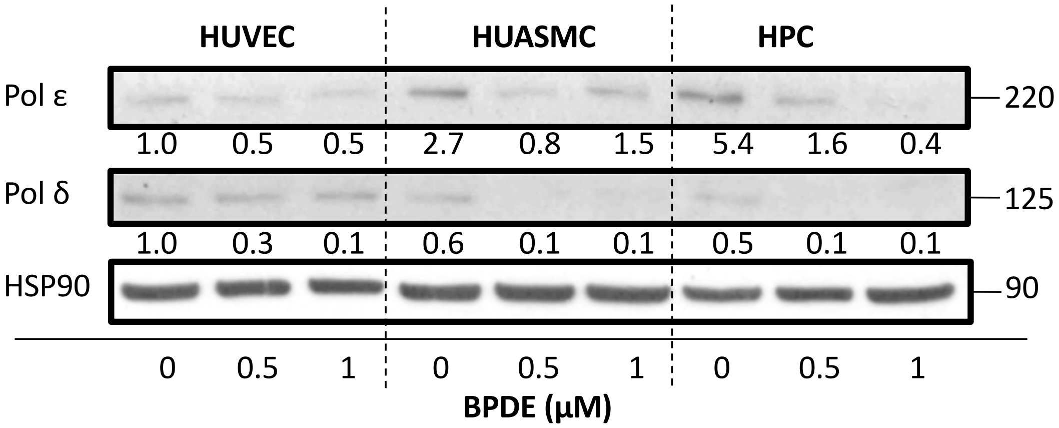

Figure S3: Expression of NER-polymerases in HUVEC, HUASMC and HPC. Exponentially growing cells were not treated or treated with BPDE with doses of 0.5 and 1 µM and cells were harvested 24 h later. HSP90 served as loading control.

Supplementary Table 1: Primer sequences for qPCR.

Gene	Forward (5`-3`)	Reverse (3`-5`)
DDB2	TGTAGCCTGGATGTGTTCT	GCATTCTGAGATTCCAAAGC
XPC	ACACCTACTACCTCTCAA	TAAATAGCAAATCTCCTTTCC
p21	TACATCTTCTGCCTTAGT	TCTTAGGAACCTCTCATT
Gapdh	CATGAGAAGTATGACAACAG	ATGAGTCCTTCCACGATA
Actb	TGGCATCCACGAAACTACC	GTGTTGGCGTACAGGTCTT
